# Language Impairment in Vascular Dementia: A Clinical Review

**DOI:** 10.1177/08919887231195225

**Published:** 2023-08-08

**Authors:** Joël Macoir

**Affiliations:** 1 Département de réadaptation, Faculté de médecine, 4440 Université Laval, Québec, QC, Canada; 2 Centre de Recherche CERVO – Brain Research Centre, Québec, QC, Canada

**Keywords:** cognitive impairment, dementia, neuropsychological testing, vascular dementia

## Abstract

Vascular cognitive impairment (VCI) encompasses a wide range of conditions, including cognitive impairment associated with stroke or vascular brain injury, mild vascular cognitive impairment, and vascular dementia (VD). Knowledge of language impairment associated with VD is far less extensive than that of Alzheimer's disease. Although not prevalent in VD, impairment in language skills has been reported. A better understanding of the neurolinguistic features associated with the different presentations of VD could facilitate medical diagnosis. In this article, we report data on language impairment in VD, with particular attention to their primary or secondary functional origin. To better appreciate this functional origin, we also outline the main characteristics of impairment in other cognitive functions. Key elements that should be considered in the speech-language assessment of individuals with possible or proven VD are also highlighted.

## Introduction

The increase in life expectancy and demographic aging, which are particularly pronounced in industrialized societies, are associated with a significant increase in major neurocognitive disorders (MNCD).^1^ Although Alzheimer’s disease (AD) is the most diagnosed cause of cognitive dysfunction in the elderly, cognitive impairment caused by vascular disease also contributes strongly to the incidence of MNCD.^2^

The prevalence of MNCD increases dramatically with age (2%-3% in people aged 70-75 years; 20%-25% in people aged 85 years or older) in both men and women, doubling approximately every 5 years between the ages of 50 and 80 years.^3^ While AD is the most common type of dementia in Western countries, accounting for approximately 60% of all cases, vascular dementia (VD) ranks second, accounting for approximately 20%-30% of all cases.^2^ The likelihood of developing dementia is slightly higher in women than in men, especially in old age, mainly due to an increased risk of being affected by AD.^4^ Conversely, VD, as well as stroke and other atherosclerotic cardiovascular disease, are more common in men than in women.^5^ This article discusses language disorders in vascular dementia and their relation to other cognitive deficits. Before turning to the topic of the article, we will first introduce the diagnostic criteria of vascular dementia and its main features.

## Diagnostic Criteria and Clinical Characteristics of Vascular Dementia

In 2011, Gorelick et al^6^ published a scientific and clinical update on the role of vascular disease in the development of cognitive impairment and dementia. According to this update, vascular cognitive impairment (VCI) encompasses a broad spectrum of conditions, including cognitive impairment associated with stroke or vascular brain injury, mild vascular cognitive impairment, and VD. As specified in this statement, it is now recognized that cerebrovascular diseases can be the cause of mild cognitive deficits that can affect several cognitive functions. The general diagnostic criteria for VD are relatively similar to those proposed in the DSM-V for the diagnosis of MNCD. As shown in Table 1, a diagnosis of probable VD involves meeting these general criteria as well as criteria related to the relationship between cognitive impairment and vascular disease.^7^

**Table 1.** Gorelick et al. (2011) Criteria for Clinical Diagnosis of Vascular Dementia and Probable Vascular Dementia.

General diagnostic criteria for VD
1. Diagnosis is based on cognitive decline from a previous baseline and a performance deficit in at least 2 cognitive domains.
2. Sufficient cognitive decline to interfere with activities of daily living
3. Diagnosis is based on tests examining at least 4 cognitive domains: Executive functions and attention, memory, language, and visuospatial functions
4. Deficits in activities of daily living are independent of motor or sensory sequelae of the vascular incident
Diagnostic criteria for probable VD
1. Presence of the general criteria for vascular dementia
2. Presence of cognitive impairment and signs of vascular disease on brain imaging and:
• Clear temporal relationship between a vascular event (e.g., stroke) and the onset of cognitive deficits
OR
• Clear association between the severity and type of cognitive impairment and the presence of diffuse subcortical cerebrovascular disease (e.g., autosomal dominant cerebral arteriopathy with subcortical infarcts and leukoencephalopathy).
3. Absence of a history of progressive cognitive impairment before or after stroke suggestive of a non-vascular neurodegenerative disease.

VD may coexist with multiple brain and systemic diseases that can impair cognition in the elderly, particularly AD.^8^ Therefore, it is often difficult to determine whether the observed cognitive decline is related solely to vascular factors or whether it is also due to AD or other underlying brain pathology. The risk factors for VD are numerous and many of them cannot be modified, such as advancing age and the presence of genetic factors. However, other factors related to lifestyle (e.g., diet, physical activity, obesity, alcohol, and tobacco use) or physiological conditions (e.g., hypertension, diabetes, hypercholesterolemia) can be modified by appropriate medical care.^9^

**Figure 1.**
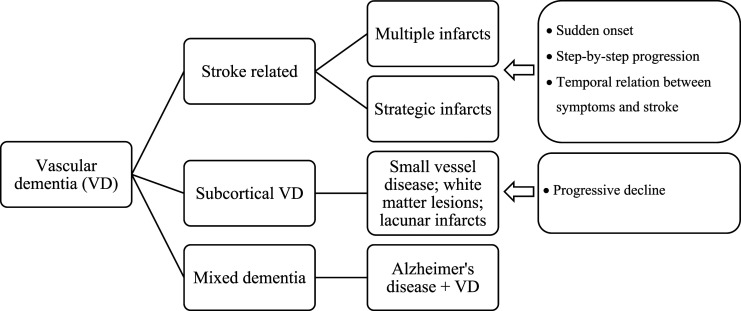
The different subtypes of vascular dementia and their neuropathological aspects.

Brain abnormalities of an arterial nature, whether age-related or pathological, can lead to cognitive dysfunction because cerebrovascular damage reduces blood supply to the brain. VCI can thus result from a number of causes, including thrombotic occlusion of large vessels with subsequent chronic cerebral hypoperfusion, cerebral emboli from carotid plaques, and dysregulation of blood pressure that compromises the integrity of the blood-blood barrier.^10^ Cerebral autosomal dominant arteriopathy with subcortical infarcts and leukoencephalopathy (CADASIL) is a hereditary disease of the small cerebral vessels that affects middle-aged adults and leads to subcortical VCI and VD.^11^ According to literature data, stroke doubles the risk of developing VD, and this risk is highest immediately after stroke.^12^ According to Leys et al.,^12^ after a stroke, approximately 30% of survivors experience dementia, and the occurrence of new-onset dementia following a stroke rises from 7% within the first year to 48% over a span of 25 years. Thus, stroke is associated with an overall decline in cognitive function and its accelerated and sustained deterioration in the years following onset.^13,14^ In addition, lacunar infarcts, microinfarcts, hemorrhages, microcerebral hemorrhages, and white matter lesions may be the result of small cerebral vessel disease.^6^ Clinically, three main subtypes of VD are generally distinguished (see Figure 1), the first two of which are directly associated with the occurrence of stroke (“stroke-related” VD): 1) the cortical VD, resulting from multiple infarcts (VDmi) affecting the cortical gray matter and often also the deep white matter; 2) The VD as a result of stroke; 3) subcortical VD, the most common, also called subcortical ischemic vascular dementia.^15^

This article addresses the impairment of language abilities in VD. As will be shown, much of this impairment results from cognitive deficits, which will therefore also be briefly addressed at the beginning of each of the following sections.

## Cognitive and Language Disorders in Vascular Dementia

Knowledge of cognitive disorders associated with VD is far less extensive than that available for AD. The most commonly described cognitive disorders in VD are bradyphrenia (slowing of mental functioning), memory impairment, dysexecutive symptoms, and attention disorders.^16^ However, the great heterogeneity in the etiology of vascular damage makes it very difficult to establish a typical profile of cognitive impairment in VD, especially since a large proportion of patients also have associated AD pathology.^8^ Therefore, depending on the location and size of the cortical and subcortical vascular lesions, a wide variety of cognitive impairment can be observed.^17^

There are very few studies that systematically describe language disorders in VD. Most of them were conducted with the aim of distinguishing VD from AD. The results of many of these studies suggest that there are no significant language differences between the two syndromes (e.g., Looi & Sachdev, 1999).^18^ However, other authors have reported conflicting results. For example, some have reported that patients with VD perform better on picture naming tasks than people with AD, whereas others have found no difference between the groups.^19,20^ However, a more nuanced picture emerges when the etiology of VD is considered in characterizing cognitive and language impairment. A summary of the main cognitive and language impairments in the 3 major subtypes of VD is presented in Table 2.

**Table 2.** Main Characteristics of Cognitive and Language Disorders in the Different Forms of Vascular Dementia.

VD subtype	Impaired cognitive functions	Impaired language abilities
VDmi	• Executive functions	• Word finding
	• Information processing speed	• Understanding of instructions
	• Episodic memory	• Possible typical aphasia profile according to brain areas affected
	• Semantic memory	• Impact of executive disorders
Post-stroke VD	Angular gyrus	Angular gyrus
	• Gerstmann syndrome	• Gerstmann syndrome
	• Praxis	• Comprehension
	Thalamus	• Reading and writing
	• Executive functions	Thalamus
	• Episodic memory	• Impact of executive disorders on lexical-semantic, syntactic and morphological processes
	Caudate nucleus	• Possible thalamic aphasia
	• Executive functions	Caudate nucleus
	Medial temporal lobe	• Non-fluent aphasia: Reduced verbal fluency, agrammatism, repetition deficit, word-finding difficulty
	• Executive functions	
	• Episodic memory	
Subcortical VD	• Psychomotor speed	Contrast between tests with and without time pressure (e.g., picture naming vs verbal fluency).
	• Executive functions	
	• With the disease progression: Episodic memory, visuospatial abilities, reasoning.	

VD: Vascular Dementia; VDmi: VD resulting from multiple infarcts.

### Language Impairment in Cortical Vascular Dementia Due to Multiple Infarcts (VDmi)

VDmi is the prevailing form of vascular dementia characterized by numerous large or small cerebral infarcts affecting cortical regions. Typically, occlusion of cerebral arteries, often caused by atherosclerotic thrombosis or cardiogenic embolism, is responsible for this condition.^21^ Cognitive disorders associated with VDim have been poorly studied. However, detection of these infarcts is important because their number seems to be a good predictor of cognitive decline in pathological aging.^22^ In the few studies published on this topic, the authors essentially report the presence of disorders affecting executive functions, speed of information processing, episodic memory, and semantic memory.^23,24^

As with other cognitive functions, very few studies have focused specifically on language disorders associated with VDmi. In most of them, the authors compared the performance of patients with VDmi to that of patients with AD.^25,26^ Compared with patients with AD, people with VDmi produce less variety and fewer words, their sentences are shorter and less syntactically complex, and their ability to name pictures is less impaired.^25,26^ In a comparative study of language, Kontiola et al^27^ showed that people affected by AD have difficulty especially in the tasks of comprehension and production of syntactically complex sentences, whereas the performance of people suffering from VDmi is impaired especially in the tasks of word recognition, naming, and repetition. In a similar study, Padovani et al^16^ showed that the performance of people with VDmi on the tasks of understanding instructions, naming pictures, and orthographic or semantic verbal fluency was significantly lower than that of neurologically healthy people. In addition, their scores were respectively higher and lower than those of patients suffering from AD in the tasks of comprehension of instructions and verbal fluency. However, not all studies have yielded supportive evidence regarding the utility of language differences in distinguishing between VDmi and AD. For example, studies by Fischer et al. and Loewenstein et al. ^28,29^ found no significant differences between the two groups on tests assessing semantic knowledge and verbal fluency. Finally, clinical profiles of aphasia can also be observed quite frequently in VDmi when infarcts lead to the dysfunction of cerebral language areas.^30,31^

### Language Impairment in Vascular Dementia Due to Stroke

It is very difficult to establish a typical cognitive profile for patients with post-stroke VD because the domains affected can vary greatly depending on the type, extent, location, and severity of the stroke. Regarding the type of stroke, patients with ischemic stroke generally have higher survival rates than patients with hemorrhagic stroke.^32^ Cerebral locations considered critical for the development of VD after stroke include the dominant hemisphere and lesions affecting the prefrontal-subcortical network, which is heavily involved in executive functions.^33^ When the initial lesion instead affects the frontal lobe, cognitive functions such as information processing speed, working memory, and executive functions are affected. The likelihood of dementia is higher in cases of multifocal infarcts compared to single infarcts or multiple infarcts occurring in a single area.^34^ However, a single large cortical-subcortical cerebral ischemic lesion, termed “strategic,” can lead to cognitive decline and VD if it is located in a functionally critical area for global cognition, but also cognitive domains such as memory and executive functions.^35-37^ Post-stroke aphasia has been the subject of numerous studies dealing with its characterization and treatment. In comparison, knowledge about language disorders in VD due to stroke is much less extensive. Nevertheless, a “strategic” ischemic lesion can also lead to deterioration of language functions, including typical aphasias, if located in a functionally critical zone for language.^38^ The exact mechanism by which a strategic single infarct leads to dementia remains incompletely understood. However, it is thought to be due to disruption of frontal-subcortical circuitry.^39^ Thus, VD due to strategic infarcts has been attributed to localizations in the angular gyrus, thalamus, caudate nucleus, and medial temporal lobe.^12,40^

The left angular gyrus is involved in various aspects of semantic processing, including concept retrieval and integration.^41,42^ This structure is also involved in other cognitive functions, such as memory retrieval, language (reading, writing, comprehension), numerical processing, spatial cognition, attention, and motor planning.^43^ Strategic infarcts of the left angular gyrus can cause Gerstmann syndrome (acalculia, right-left disorientation, agraphia, digital agnosia) as well as disorders of language and praxis.^44,45^

The role of the thalamus, which is neuroanatomically connected to the dorsolateral prefrontal cortex, extends beyond relaying sensory information to the cortex. It also serves as a mediator for interactions between higher-level cognitive processes, such as executive functions, attention, memory, language, and sensorimotor functions.^46^ When strategic infarcts affect the right and or the left thalamus, cognitive damage mainly affects executive functions,^47^ but also episodic memory,^48^ praxis,^49^ and language. The left thalamus also plays an important role in controlling lexical-semantic, syntactic, and morphological language processes.^50^ Thus, left thalamic infarction may be the cause of fluent aphasia, sometimes referred to as “thalamic” aphasia, characterized by mild comprehension and repetition deficits, anomia, and reading and writing impairments.^51,52^

Cognitive dysfunction affecting executive functions and language is also observed in the sequelae of vascular lesions of the caudate nucleus.^53^ This subcortical structure contains numerous clusters of neurons that establish functional connections with cortical areas, forming integral components of distributed networks engaged in working and episodic memory, executive functions.^54,55^ The caudate nucleus is also known to play a role in language control, particularly in the inhibition and selection of linguistic representations.^56^ Infarcts of the left caudate nucleus can result in language impairments similar to those of nonfluent aphasia, characterized by reduced fluency, agrammatism, impaired repetition, and word-finding difficulty.^57^ However, this clinical picture of language disorders due to caudate nucleus lesions is not exclusive, as other manifestations are also possible, such as the production of semantic paraphasias in conversational speech or aphasia of the transcortical sensory type, characterized by a fluid and abundant spontaneous language but affected by semantic paraphasias and ideational inconsistencies.^58,59^

The bilateral medial temporal lobe is known to play an essential role in episodic memory.^60^ Atrophy of the medial temporal lobe as a result of stroke is relatively common^61^ and appears to be associated with an increased risk of developing subsequent VD.^62^ Associated cognitive impairment includes episodic memory, as well as executive functions.^63,64^ The data in the current literature do not allow us to decide on the presence or even the existence of language disorders in VD due to atrophy of the medial temporal lobe after stroke.

### Language Impairment in Subcortical Vascular Dementia

When vascular damage is subcortical, the cognitive picture of VD is more homogeneous and dominated mainly by psychomotor slowing and executive dysfunction caused by lacunar infarcts affecting fronto-subcortical structures and circuits.^65^ Specifically, subcortical vascular dementia showed a greater reduction in connections within the orbitofrontal cortex and dorsolateral prefrontal cortex.^66^ These regions, known as the main components of the prefrontal/subcortical circuit, were found to be disrupted, leading to executive dysfunction.^67^ As the disease progresses and deficits multiply, other cognitive functions are gradually impaired, including semantic memory and episodic memory.^23^ However, compared to AD, the episodic memory impairments observed in VD are explained by executive difficulties in retrieving information from long-term memory rather than by impaired encoding.^68^ In CADASIL cognitive disruptions span a spectrum from mild cognitive slowing to impairment of executive functions, which can lead to widespread decline in cognitive performance and to dementia.^69,70^ The slowing of processing speed that marks the onset of the disease is usually followed by impaired attention and executive functions.^71,72^ As the disease progresses, episodic memory may also decline, as may reasoning and visuospatial abilities.^73,74^

Early onset and progressive deterioration of language impairment without evidence of focal lesions on brain imaging are among the features that make the diagnosis of subcortical VD less likely.^75^ Thus, similar to other cognitive functions, language impairments in the subcortical VD are not primary in nature but result from impairment in executive functions.^76^ In comparative studies with AD, the poorer performance of people with subcortical VD on fluency tasks whereas they perform better on picture naming suggests an executive origin of their language impairments.^27,77^ Qualitative analysis of the errors (i.e., categorical semantic paraphasias) that people with subcortical VD make in picture naming,^20^ or the semantic grouping strategies they use in verbal fluency,^78^ also reflect the general preservation of their lexical and semantic knowledge.^76^ Finally, the slowing of information processing due to the disruption of the cerebral white matter network characteristic of the subcortical VD also affects linguistic abilities, especially on tests with time constraints (e.g., verbal fluency).^79,80^

Knowledge about language abilities in CADASIL is very limited. Some studies have reported preservation of these abilities, while others have shown impairment, but without specifying the language areas affected.^72,81,82^ The same observation emerges from single-case studies of participants with CADASIL, with some indicating impairment of language functions (expressive language) and others tending to report their preservation.^83-85^

## The Assessment of Language in Vascular Dementia

Unlike MNCD such as primary progressive aphasia, VD is not characterized by specific and typical deficits in language functions. As for other MNCD, language assessment alone cannot diagnose VD, especially because of the secondary functional origin of most language impairments (i.e., primary deficits in executive functions and psychomotor speed). The wide heterogeneity of clinical profiles associated with VD makes it more difficult to establish a typical neurolinguistic profile. As described in this article, the nature of language disorders depends strongly on their etiology. According to data from the scientific literature, all facets of language function may be impaired in individuals with cortical VD resulting from multiple infarcts or due to stroke. Therefore, a comprehensive speech-language assessment should be performed in this clinical population.

Language disorders in the subcortical VD are more homogeneous and result essentially from executive dysfunction and psychomotor slowing, which dominate the cognitive picture. Accordingly, speech-language pathology assessment should focus on tasks and tests to contrast the involvement of executive functions. For example, differential performance on comprehension of syntactically simple vs complex sentences of the same length would allow objectification of the executive contribution to comprehension disorders. Similarly, the identification of word-finding difficulty in spontaneous speech without significant anomia in picture naming would strongly suggest a secondary language disorder of executive origin. Finally, psychomotor slowing is likely to be objectified in all language tasks, although to a greater extent in tasks performed under time pressure, such as verbal fluency.

## Conclusion

VD encompass a wide spectrum of pathologies due to cerebrovascular damage of various etiologies. They are almost always language disorders, but their extent, nature, and functional origin can vary widely. There is a substantial overlap of language impairment in cortical VD, due to multiple infarcts and AD. At present, the data available in the literature do not allow us to identify the clinical signs that make it possible to distinguish the two pathologies. The objectification of clinical pictures related to “classic” aphasias is also more compatible with the post-stroke VD, although they can also be observed in cortical VD resulting from multiple infarcts. Likewise, these neurolinguistic profiles are markedly different from those typically associated with most MNCD, with the exception of those observed in primary progressive aphasia. Finally, the more homogeneous nature of the language impairments associated with subcortical VD, which are also very typical of this pathology, allows for a greater contribution of speech-language assessment to the differential diagnosis of VD.

There are still very few studies that specifically address oral and written language disorders in VD. Large gaps in knowledge remain to be filled, especially regarding neurolinguistic disorders associated with cortical VD, resulting from multiple infarcts. Characterization of language disorders is obviously important, but identification of the underlying functional origin is equally important, whether for improving differential diagnosis or for developing therapeutic approaches adapted to this clinical population.
